# The cumulative effect of reporting and citation biases on the apparent efficacy of treatments: the case of depression

**DOI:** 10.1017/S0033291718001873

**Published:** 2018-08-02

**Authors:** Y. A. de Vries, A. M. Roest, P. de Jonge, P. Cuijpers, M. R. Munafò, J. A. Bastiaansen

**Affiliations:** 1 Department of Psychiatry, Interdisciplinary Center Psychopathology and Emotion regulation, University of Groningen, University Medical Center Groningen, Groningen, the Netherlands; 2 Developmental Psychology, Department of Psychology, University of Groningen, Groningen, the Netherlands; 3 Department of Clinical, Neuro-, and Developmental Psychology, Amsterdam Public Health Research Institute, Vrije Universiteit Amsterdam, Amsterdam, the Netherlands; 4 MRC Integrative Epidemiology Unit, University of Bristol, Bristol, UK; 5 UK Centre for Tobacco and Alcohol Studies, School of Experimental Psychology, University of Bristol, Bristol, UK; 6 Department of Education and Research, Friesland Mental Health Care Services, Leeuwarden, The Netherlands

**Keywords:** Antidepressants, bias, citation bias, depression, psychotherapy, reporting bias

## Introduction

Evidence-based medicine is the cornerstone of clinical practice, but it is dependent on the quality of evidence upon which it is based. Unfortunately, up to half of all randomized controlled trials (RCTs) have never been published, and trials with statistically significant findings are more likely to be published than those without (Dwan *et al*., 2013). Importantly, negative trials face additional hurdles beyond study publication bias that can result in the disappearance of non-significant results (Boutron *et al*., 2010; Dwan *et al*., 2013; Duyx *et al*., 2017). Here, we analyze the cumulative impact of biases on apparent efficacy, and discuss possible remedies, using the evidence base for two effective treatments for depression: antidepressants and psychotherapy.

## Reporting and citation biases

We distinguish among four major biases, although others exist: study publication bias, outcome reporting bias, spin, and citation bias. While study publication bias involves non-publication of an entire study, outcome reporting bias refers to non-publication of negative outcomes within a published article or to switching the status of (non-significant) primary and (significant) secondary outcomes (Dwan *et al*., 2013). Both biases pose an important threat to the validity of meta-analyses (Kicinski, 2014).

Trials that faithfully report non-significant results will yield accurate effect size estimates, but results interpretation can still be positively biased, which may affect *apparent* efficacy. Reporting strategies that could distort the interpretation of results and mislead readers are defined as spin (Boutron *et al*., 2010). Spin occurs when authors conclude that the treatment is effective despite non-significant results on the primary outcome, for instance by focusing on statistically significant, but secondary, analyses (e.g. instead of concluding that treatment X was not more *effective* than placebo, concluding that treatment X was *well tolerated* and was effective *in patients who had not received prior therapy*). If an article has been spun, treatments are perceived as more beneficial (Boutron *et al*., 2014). Finally, citation bias is an obstacle to ensuring that negative findings are discoverable. Studies with positive results receive more citations than negative studies (Duyx *et al*., 2017), leading to a heightened visibility of positive results.

## The evidence base for antidepressants

We assembled a cohort of 105 depression trials, of which 74 were also included in a previous study on publication bias (Turner *et al*., 2008); we added 31 trials of novel antidepressants (approved after 2008) from the Food and Drug Administration (FDA) database (see online Supplementary materials). Pharmaceutical companies must preregister all trials they intend to use to obtain FDA approval; hence, trials with non-significant results, even if unpublished, are still accessible.

**Fig. 1.**
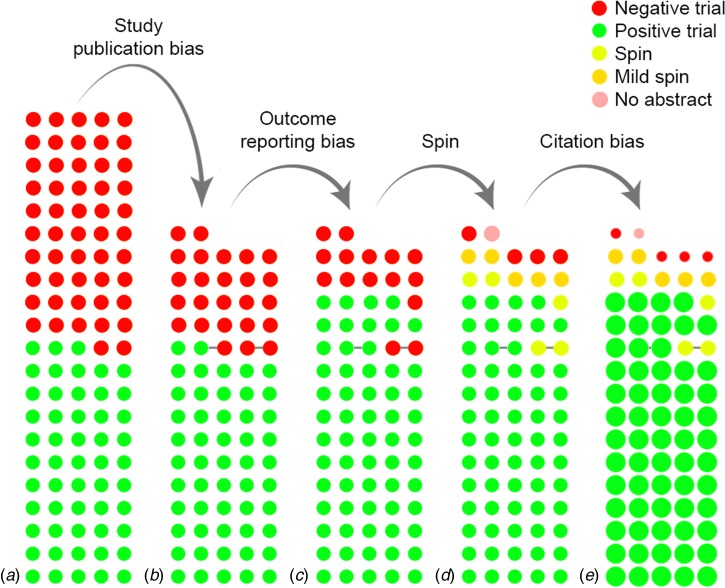
The cumulative impact of reporting and citation biases on the evidence base for antidepressants. (*a*) displays the initial, complete cohort of trials, while (*b*) through (*e*) show the cumulative effect of biases. Each circle indicates a trial, while the color indicates the results or the presence of spin. Circles connected by a grey line indicate trials that were published together in a pooled publication. In (*e*), the size of the circle indicates the (relative) number of citations received by that category of studies.

Figure 1 demonstrates the cumulative impact of reporting and citation biases. Of 105 antidepressant trials, 53 (50%) trials were considered positive by the FDA and 52 (50%) were considered negative or questionable (Fig. 1*a*). While all but one of the positive trials (98%) were published, only 25 (48%) of the negative trials were published. Hence, 77 trials were published, of which 25 (32%) were negative (Fig. 1*b*). Ten negative trials, however, became ‘positive’ in the published literature, by omitting unfavorable outcomes or switching the status of the primary and secondary outcomes (Fig. 1*c*). Without access to the FDA reviews, it would not have been possible to conclude that these trials, when analyzed according to protocol, were *not* positive. Among the remaining 15 (19%) negative trials, five were published with spin in the abstract (i.e. concluding that the treatment was effective). For instance, one article reported non-significant results for the primary outcome (*p* = 0.10), yet concluded that the trial ‘demonstrates an antidepressant effect for fluoxetine that is significantly more marked than the effect produced by placebo’ (Rickels *et al*., 1986). Five additional articles contained mild spin (e.g. suggesting the treatment is at least numerically better than placebo). One article lacked an abstract, but the discussion section concluded that there was a ‘trend for efficacy’. Hence, only four (5%) of 77 published trials unambiguously reported that the treatment was not more effective than placebo in that particular trial (Fig. 1*d*). Compounding the problem, positive trials were cited three times as frequently as negative trials (92 *v.* 32 citations in Web of Science, January 2016, *p* < 0.001, see online Supplementary material for further details) (Fig. 1*e*). Among negative trials, those with (mild) spin in the abstract received an average of 36 citations, while those with a clearly negative abstract received 25 citations. While this might suggest a synergistic effect between spin and citation biases, where negatively presented negative studies receive especially few citations (de Vries *et al*., 2016), this difference was not statistically significant (*p* = 0.50), likely due to the small sample size. Altogether, these results show that the effects of different biases accumulate to hide non-significant results from view.

## The evidence base for psychotherapy

While the pharmaceutical industry has a financial motive for suppressing unfavorable results, these biases are also present in the other areas of research, such as psychotherapy. Without a standardized trial registry, however, they are more difficult to detect and disentangle. Statistical tests suggest an excess of positive findings in the psychotherapy literature, due to either study publication bias or outcome reporting bias (Flint *et al*., 2015). Of 55 National Institutes of Health-funded psychotherapy trials, 13 (24%) remained unpublished (Driessen *et al*., 2015), and these had a markedly lower effect size than the published trials.

Regarding spin, 49 (35%) of 142 papers were considered negative in a recent meta-analysis (Flint *et al*., 2015), but we found that only 12 (8%) abstracts concluded that psychotherapy was not more effective than a control condition. The remaining abstracts were either positive (73%) or mixed (19%) (e.g. concluding that the treatment was effective for one outcome but not another). Although we could not establish the pre-specified primary outcome for these trials, and therefore cannot determine whether a specific abstract is biased, published psychotherapy trials, as a whole, clearly provide a more positive impression of the effectiveness of psychotherapy than is justified by available evidence. Positive psychotherapy trials were also cited nearly twice as frequently as negative trials (111 citations *v.* 58, *p* = 0.003). Negative trials with a positive or mixed abstract were cited more often than those with a negative abstract (59 and 87 citations, respectively *v.* 26, *p* = 0.05); however, the small sample size precludes definitive conclusions on the effects of spin on citation rates.

## Preventing bias

Mandatory prospective registration has long been advocated as a solution for study publication and outcome reporting bias. The International Committee of Medical Journal Editors (ICMJE) began requiring prospective registration of clinical trials as a precondition for publication in 2005, but many journals do not require registration (Knüppel *et al*., 2013) and others allow retrospective registration (Harriman and Patel, 2016). Since 2007, the FDA also requires prospective registration of most drug trials. This increasing pressure may explain why recently completed, negative antidepressant trials are more frequently published than older negative trials: all negative trials that remained unpublished were completed before 2004, while the 25 trials completed in 2004 or later (including 14 for which registration was legally required) were all published, even though nine were negative. A regulatory requirement is likely to be one of the most effective measures to ensure universal registration; unfortunately, the 2007 law excludes trials of behavioral interventions (e.g. psychotherapy) and phase 1 (healthy volunteer) trials.

Nevertheless, registration seems insufficient to ensure complete and accurate reporting of a trial. Only around half of all trials registered at ClinicalTrials.gov were published within two years of completion (Ross *et al*., 2009), and non-reporting of protocol-specified outcomes or the silent addition of new outcomes is also common (Jones *et al*., 2015, http://www.compare-trials.org). Close examination of registries by independent researchers may be necessary for registration to be a truly effective deterrent to study publication and outcome reporting bias. An alternative (or addition) to registration could be publication of study protocols or ‘registered reports’, in which journals accept a study for publication based on the introduction and methods, before the results are known. Widespread adoption of this format might also help to prevent spin, by reducing the pressure that researchers might feel to ‘oversell’ their results to get published. Furthermore, in our analysis, positive studies were published in journals with a higher median impact factor (and thus higher visibility) than negative studies (3.5 *v.* 2.4 for antidepressant trials and 3.1 *v.* 2.6 for psychotherapy trials), which may be one driver behind the difference in citation rates. Hence, adoption of registered reports might also reduce citation bias by reducing the tendency for positive studies to be published in higher impact journals. Peer reviewers could also play a crucial role in ensuring that abstracts accurately report trial results and that important negative studies are cited. Finally, the prevalence of spin and citation biases also shows the importance of assessing a study's actual results (rather than relying on the authors’ conclusions) and of conducting independent literature searches, since reference lists may yield a disproportionate number of positive (and positively presented) studies.

## Conclusions

The problem of study publication bias is well-known. Our examination of antidepressant trials, however, shows the pernicious cumulative effect of additional reporting and citation biases, which together eliminated most negative results from the antidepressant literature and left the few published negative results difficult to discover. These biases are unlikely to be unique to antidepressant trials. We have already shown that similar processes, though more difficult to assess, occur within the psychotherapy literature, and it seems likely that the effect of these biases accumulates whenever they are present. Consequently, researchers and clinicians across medical fields must be aware of the potential for bias to distort apparent treatment efficacy, which poses a threat to the practice of evidence-based medicine.

## Supplementary material

For supplementary material accompanying this paper visit http://dx.doi.org/10.1017/S0033291718001873. click here to view supplementary material
