# Supplementary material for: The cumulative effect of reporting and citation biases on the apparent efficacy of treatments: the case of depression
Source: Psychol Med. 2018 Aug 2;48(15):2453–5. doi: 10.1017/S0033291718001873 (PMC6190062; doi:10.1017/S0033291718001873)
Supplement: Supplementary file 1 [file S0033291718001873sup001.docx]

**Supplemental materials**

All data extraction and coding took place between November 2015 and February 2016.

**Antidepressant trials**

We included all trials included in the study by Turner *et al*. (2008). Data on p-values provided by the Food and Drug Administration and by the matched journal articles (if the trial was published) were extracted from Appendix Table A. We based our coding of the outcome of the trial and of the matching publication as positive or negative on these p-values.

In the course of performing searches for another study (in preparation), we found an additional short report (Miller *et al.* 1989), which matched trial UK-06 of paroxetine. Hence, we included this trial as ‘published’, although it is listed as unpublished in the original study by Turner *et al*. (2008).

Four antidepressants were approved by the FDA since Turner *et al.*’s work: desvenlafaxine, vilazodone, vortioxetine, and levomilnacipran. For these antidepressants, we downloaded the FDA drug application packages (a.k.a. reviews) from the Drugs@FDA website, following the approach described by Turner (2013).

We included all phase 2 and 3 trials reviewed in these application packages. Two independent raters coded the FDA conclusion for each trial as positive, negative, or questionable. Trial conclusion was considered questionable if the trial had ‘failed’, i.e. if neither the investigative antidepressant nor an active comparator performed better than placebo, consistent with Turner *et al.*’s (2008) coding. Trials coded as negative or questionable were later grouped together as ‘negative trials’.

To match these trials to journal publications, we searched for matching publications on Embase, PubMed, and Cochrane Central. We searched for the drug name in the title of the publication and the word “placebo” in all fields. Matches were confirmed by comparing the following variables: trial duration, dosage groups, active comparator (if used), primary outcome, sample size, and baseline, endpoint, and change scores on the primary outcome. If our search yielded no matching publication for a trial, we searched for recent (systematic) reviews of the antidepressant in question and examined all trials included in the review. Consistent with Turner *et al.* trial (2008) outcome according to the journal article was determined by examining the p-value associated with the outcome identified as primary in the methods or results section or, if no outcome was identified as primary, by examining the outcome mentioned first in the results section. The article outcome was considered positive if this outcome was statistically significant (p < 0.05) and as negative otherwise. Coding was done in duplicate by two independent raters.

Abstract coding for papers with positive outcomes was performed by a single rater, while abstract coding for all negative papers was performed by two independent raters. Abstracts were considered positive if the abstract concluded that the antidepressant was effective (i.e. “spin” in case of a negative paper); questionable if the abstract concluded that the antidepressant was numerically (but not statistically significantly) better than placebo or that the trial results could not be interpreted because the active comparator also failed to separate from placebo (“mild spin”); and negative if the abstract concluded that the antidepressant did not show efficacy in this particular trial (“no spin”). Agreement between raters was good.

**Psychotherapy trials**

We initially included all 149 published trials included in the study by Flint *et al.* (2015). However, we excluded trials that were only published in a dissertation or book and trials that were not published in English, since non-English language articles are likely to be cited less frequently. We also excluded articles which either did not have an abstract or for which the abstract did not discuss the results of the trial. Consequently, we included 142 trials in our analyses.

We coded the outcome of the trial based upon the p-value for the trial as listed in Supplementary Table S1 of Flint *et al.* (2015). All abstracts were coded by two independent raters. Abstracts were coded as positive, negative, or mixed (if it mentioned some positive and some negative outcomes). Agreement between raters was considered good (κ = 0.66 and weighted κ = 0.74).

**Citations**

We looked up the number of citations to all included psychotherapy and antidepressant trials on Web of Science (Core Collection, January 2016). We excluded articles without abstracts and articles that could not be located in Web of Science. Given the non-normal distribution of citations, we performed non-parametric Mann-Whitney tests (in R 3.4.0) to test for a difference between articles with negative or positive outcomes, and between negative articles with (mild) spin and those without spin.

Our main analyses examined all citations to a trial since publication (see supplemental tables 1 and 2). In a secondary analysis, we included only trials published in 2011 or earlier and examined the number of citations in the five years immediately following publication. For this secondary analysis, we accounted for the growth in scientific output (and hence the growing opportunity to be cited) by adjusting the number of citations for the number of papers published per year. To do this, we calculated an adjustment factor by dividing the number of papers indexed in the category Psychology or Psychiatry in Web of Science per year by the number indexed in 1977. The number of citations received by a given trial in a given year was subsequently divided by this adjustment factor. Results were comparable to our main analyses (see supplemental tables 3 and 4).

**Impact factors**

To compare the impact factors of the journals that positive vs. negative studies were published in, we looked up the impact factor of each journal in the year of publication of the study on Web of Science’s Journal Citation Reports. If no impact factor was available for the year of publication, we took the impact factor from the closest year. This was particularly the case for older studies, published before 1997, as the Journal Citation Reports database has no information prior to 1997. Because impact factors have increased over the years and negative studies tended to be published later than positive studies, we also compared the 2016 impact factors between positive and negative studies as a sensitivity analysis, which yielded similar results.

**References**

**Flint J, Cuijpers P, Horder J, Koole SL, Munafò MR** (2015). Is there an excess of significant findings in published studies of psychotherapy for depression? *Psychological Medicine* **45**, 439–446.

**Miller SM, Naylor GJ, Murtagh M, Winslow G** (1989). A double-blind comparison of paroxetine and placebo in the treatment of depressed patients in a psychiatric outpatient clinic. *Acta Psychiatrica Scandinavica* **supp. 350**, 143–144.

**Turner EH** (2013). How to access and process FDA drug approval packages for use in research. *BMJ*, f5992.

**Turner EH, Matthews AM, Linardatos E, Tell RA, Rosenthal R** (2008). Selective publication of antidepressant trials and its influence on apparent efficacy. *New England Journal of Medicine* **358**, 252–60.

**Supplemental tables**

**Supplemental table 1. Citations to published antidepressant trials, by article outcome and abstract.**

| **Article outcome** | **Presence of spin** | **Number of articles** | **Citations** | | |
| --- | --- | --- | --- | --- | --- |
|  |  |  | Mean (SD) | Median (IQR) | Range |
| **Positive** | **-** | 58 | 91.8 (71.8) | 63.0 (40.8 – 122.5) | 1 – 292 |
| **Negative** | **All** | 12 | 32.2 (17.0) | 35.0 (23.0 – 39.3) | 2 - 66 |
|  | **Spin** | 4 | 34.3 (23.7) | 31.0 (23.3 – 42.0) | 9 – 66 |
|  | **Mild spin** | 4 | 37.0 (10.4) | 38.5 (34.3 – 41.3) | 23 – 48 |
|  | **No spin** | 4 | 25.3 (17.1) | 29.5 (17.8 – 37.0) | 2 - 40 |

**Supplemental table 2. Citations to published psychotherapy trials, by article outcome and abstract.**

| **Article outcome** | **Abstract** | **Number of articles** | **Citations** | | |
| --- | --- | --- | --- | --- | --- |
|  |  |  | Mean (SD) | Median (IQR) | Full range |
| **Positive** | **-** | 86 | 112.6 (175.0) | 66.5 (32.0 – 137.0) | 4 – 1464 |
| **Negative** | **All** | 44 | 58.0 (65.7) | 36.5 (19.5 – 73.3) | 4 – 291 |
|  | **Positive** | 24 | 59.4 (61.5) | 39.0 (20.3 – 87.3) | 9 - 291 |
|  | **Mixed** | 10 | 86.8 (92.9) | 40.5 (33.3 – 106.5) | 10 – 282 |
|  | **Negative** | 10 | 25.7 (15.7) | 23.5 (13.3 – 37.3) | 4 – 54 |

**Supplemental table 3. Citations to published antidepressant trials in the first 5 years after publication, adjusted by scientific output per year (see methods).**

| **Article outcome** | **Presence of spin** | **Number of articles** | **Citations** | | |
| --- | --- | --- | --- | --- | --- |
|  |  |  | **Mean (SD)** | **Median (IQR)** | **Range** |
| **Positive** | **-** | 48 | 23.5 (15.6) | 16.3 (12.2 – 39.9) | 1.5 – 60.6 |
| **Negative** | **All** | 8 | 7.3 (3.4) | 7.5 (6.8 – 9.3) | 0.0 – 11.3 |
|  | **Spin** | 4 | 6.8 (4.9) | 8.0 (5.2 – 9.6) | 0.0 – 11.3 |
|  | **Mild spin** | 3 | 8.2 (1.7) | 8.1 (7.3 – 9.0) | 6.5 – 9.9 |
|  | **No spin** | 1 | 6.9 (N/A) | 6.9 (N/A) | N/A |

**Supplemental table 4. Citations to published psychotherapy trials in the first 5 years after publication, adjusted by scientific output per year (see methods).**

| **Article outcome** | **Abstract** | **Number of articles** | **Citations** | | |
| --- | --- | --- | --- | --- | --- |
|  |  |  | **Mean (SD)** | **Median (IQR)** | **Full range** |
| **Positive** | **-** | 86 | 15.5 (19.1) | 10.3 (4.9 – 18.7) | 0.8 – 141.0 |
| **Negative** | **All** | 44 | 8.9 (11.0) | 6.3 (3.2 – 9.7) | 0.0 – 60.7 |
|  | **Positive** | 24 | 8.1 (7.9) | 6.4 (4.3 – 7.8) | 1.1 – 38.6 |
|  | **Mixed** | 10 | 15.3 (18.3) | 9.7 (4.4 – 15.1) | 0.9 – 60.7 |
|  | **Negative** | 10 | 4.2 (3.8) | 3.4 (2.4 – 4.2) | 0.0 – 13.4 |
